# Transmural APD heterogeneity determines ventricular arrhythmogenesis in LQT8 syndrome: Insights from Bidomain computational modeling

**DOI:** 10.1371/journal.pone.0305248

**Published:** 2024-07-05

**Authors:** Simone Scacchi, Luca F. Pavarino, Andrea Mazzanti, Alessandro Trancuccio, Silvia G. Priori, Piero Colli Franzone

**Affiliations:** 1 Dipartimento di Matematica, Università degli Studi di Milano, Milano, Italy; 2 Dipartimento di Matematica, Università degli Studi di Pavia, Pavia, Italy; 3 Molecular Cardiology, Centro Nacional de Investigaciones Cardiovasculares (CNIC), Madrid, Spain; 4 Molecular Cardiology, IRCCS Istituti Clinici Scientifici Maugeri, Pavia, Italy; 5 Department of Molecular Medicine, University of Pavia, Pavia, Italy; University of Minnesota, UNITED STATES

## Abstract

Long QT Syndrome type 8 (LQT8) is a cardiac arrhythmic disorder associated with Timothy Syndrome, stemming from mutations in the CACNA1C gene, particularly the G406R mutation. While prior studies hint at CACNA1C mutations’ role in ventricular arrhythmia genesis, the mechanisms, especially in G406R presence, are not fully understood. This computational study explores how the G406R mutation, causing increased transmural dispersion of repolarization, induces and sustains reentrant ventricular arrhythmias. Using three-dimensional numerical simulations on an idealized left-ventricular model, integrating the Bidomain equations with the ten Tusscher-Panfilov ionic model, we observe that G406R mutation with 11% and 50% heterozygosis significantly increases transmural dispersion of repolarization. During S1-S4 stimulation protocols, these gradients facilitate conduction blocks, triggering reentrant ventricular tachycardia. Sustained reentry pathways occur only with G406R mutation at 50% heterozygosis, while neglecting transmural heterogeneities of action potential duration prevents stable reentry, regardless of G406R mutation presence.

## Introduction

Long QT syndromes (LQTS) are a group of disorders characterized by an abnormally prolonged QT interval on the electrocardiogram (ECG), leading to a high risk of life-threatening ventricular arrhythmias, such as tachycardia and fibrillation, see e.g. [1, 2]. This study focuses on LQTS type 8 (LQT8), associated with Timothy syndrome (TS), a multisystem disorder involving congenital heart disease, syndactyly, immune deficiency, and autism. TS arises from mutations in the CACNA1C gene, which encodes the CaV1.2 subunit of the L-type voltage-dependent calcium channel ICaL. Notable mutations include G406R, G402S, G1783C, P381S, M456I, A582D, and R858H, see [3–9].

Previous experimental studies have shown that CACNA1C mutations can alter ICaL, potentially triggering ventricular arrhythmias. The recent in vivo study by Sanchez et al. [10] is particularly significant, as it investigates the arrhythmogenic mechanisms in the first knock-in swine model of TS1, demonstrating that cardiac activation impairments may lead to conduction blocks and reentry induction.

Computational simulations have shown that CACNA1C mutations prolong action potential duration (APD), leading to early afterdepolarizations (EADs; see [11–15]) and increased sarcoplasmic reticulum (SR) calcium content, resulting in delayed afterdepolarizations (DADs; see [7, 14, 16, 17]). However, these studies mainly focused on single-cell simulations. Fewer studies have explored the relationship between CACNA1C mutations and arrhythmogenic substrate increase in tissue simulations, primarily considering G1911R and R858H mutations, see [18–20]. Our study aims to shed light on the G406R mutation, which is less understood in terms of its role in ventricular arrhythmias.

LQTS are linked to increased transmural and spatial dispersion of repolarization, a known precursor for reentrant ventricular tachycardia and fibrillation, [21, 22]. This dispersion is attributed to intrinsic differences in APD across the three principal cell types of the ventricular myocardium (endocardial, midmyocardial and epicardial), and to the extent to which these repolarization differences are damped by electrotonic forces, see [23–25].

Our study aims to elucidate the mechanisms by which the G406R CACNA1C mutation increases transmural dispersion of repolarization, potentially inducing and sustaining reentrant ventricular arrhythmia. We employ numerical simulations using the Bidomain model of electrocardiology [26–29], a non-linear reaction-diffusion system of Partial Differential Equations (PDEs), coupled with the ten Tusscher-Panfilov membrane model (TP06, see [30, 31]), a stiff system of Ordinary Differential Equations (ODEs), describing the ion currents through the human ventricular cellular membrane. For numerical approximation, we use finite elements in space and semi-implicit finite differences in time, accelerated using parallel codes based on advanced Domain Decomposition techniques and run on Linux clusters with up to 128 cores.

## Methods

### Mathematical models

In the numerical section, we have simulated the bioelectrical activity of a three-dimensional idealized left ventricular geometry. We model the bioelectrical activity of a three-dimensional idealized left ventricular geometry using the Bidomain representation of the cardiac tissue [26–29].

Let Ω denote the three-dimensional region occupied by the cardiac tissue. Then, according to the Bidomain model, the evolution of the transmembrane potential *v*(**x**, *t*), extracellular potential *u*_*e*_, gating variables **w**(**x**, *t*) and ionic concentrations **c**(**x**, *t*) is described by the following system of partial differential equations:

$$\begin{array}{c} \left\{ \begin{array}{cc} c_{m}\partial_{t}v-\text{div}\;\left(D_{i}\nabla v\right)-\text{div}\;\left(D_{i}\nabla u_{e}\right)+i_{ion}\left(v,\text{w},\text{c}\right)=i_{app} & \;\text{in}\;\Omega \\ \partial_{t}w-R_{w}\left(v,\text{w},\text{c}\right)=0,\;\partial_{t}c-R_{c}\left(v,\text{w},\text{c}\right)=0 & \;\text{in}\;\Omega \\ -\text{div}\;\left(D_{i}+D_{e}\right)\nabla u_{e}=\text{div}\;D_{i}\nabla v & \;\text{in}\;\Omega \\ {\text{n}}^{T}D_{i}\nabla \left(v+u_{e}\right)=0 & \;\text{on}\;\partial \Omega \\ {\text{n}}^{T}D_{e}\nabla u_{e}=0 & \;\text{on}\;\partial \Omega \end{array}\right. \end{array}$$ (1)

with appropriate initial conditions on *v*(**x**, 0), **w**(**x**, 0) and **c**(**x**, 0). Here *c*_*m*_ and *i*_*ion*_ denote the capacitance and the ionic current of the membrane per unit volume, *i*_*app*_ represents the applied current per unit volume, *D*_*i*,*e*_ are the intra- and extracellular transversely isotropic conductivity tensors.

Assuming transversely isotropic properties of the intra- and extracellular media, the conductivity tensors are given by

$$\begin{array}{c} D_{i,e}=\sigma_{t}^{i,e}\;I+\left(\sigma_{l}^{i,e}-\sigma_{t}^{i,e}\right){\text{a}}_{l}⊗{\text{a}}_{l}, \end{array}$$ (2)

where $\sigma_{l}^{i,e},\;\sigma_{t}^{i,e}$ are the conductivity coefficients of the intra- and extracellular media measured along the fiber direction **a**_*l*_ and any cross fiber direction, respectively.

The ionic current *i*_*ion*_(*v*, **w**, **c**) and the associated ordinary differential equations for the gating and ionic concentration variables are modeled according to the ten Tusscher-Panfilov membrane model (TP06) [30, 31], available from the cellML depository (models.cellml.org/cellml), consisting of an ordinary differential equations (ODEs) system with 17 ODEs modeling the main ionic currents dynamics. The TP06 is one of the most used human ionic models with biophysical details and it has been extensively validated against experimental data from various experimental conditions, including different pacing rates, drug effects, and pathological states. Of course other biophysically detailed human ionic models could be considered as well, as long as they include the L-type calcium current (*I*_*CaL*_) which in the LQT8 syndrome is altered by the CACNA1C mutation.

### Numerical methods

The space discretization of the Bidomain system is performed by employing hexahedral isoparametric *Q*_1_ finite elements, while the time discretization is based on the following double operator splitting procedure: a) split the ODEs from the PDEs and b) split the elliptic PDE from the parabolic one. The adopted numerical procedure has been described in detail in [32] and in the recent work [33]. We also refer to [34–36] for other numerical strategies adopted in the literature.

For sake of clarity, we describe here the time discretization applied to the strong formulation of the Bidomain system. Let us denote by *τ* the time step size. At each time step, given *v*^*n*^, **w**^*n*^, **c**^*n*^, we proceed as follows:

1. find **w**^*n*+1^, **c**^*n*+1^ by solving
  $$\left\{ \begin{array}{c} \frac{{\text{w}}^{n+1}-{\text{w}}^{n}}{\tau }=R_{w}\left(v^{n},{\text{w}}^{n+1},{\text{c}}^{n}\right)\\ \frac{{\text{c}}^{n+1}-{\text{c}}^{n}}{\tau }=R_{c}\left(v^{n},{\text{w}}^{n+1},{\text{c}}^{n}\right); \end{array}\right.$$
2. find $u_{e}^{n}$ by solving the elliptic PDE
  $$\begin{array}{c} -\text{div}\;\left(D_{i}+D_{e}\right)\nabla u_{e}^{n}=\text{div}\;D_{i}\nabla v^{n}; \end{array}$$ (3)
3. find *v*^*n*+1^ by solving the parabolic PDE
  $$\begin{array}{c} c_{m}\frac{v^{n+1}-v^{n}}{\tau }-\text{div}\;\left(D_{i}\nabla v^{n+1}\right)-\text{div}\;\left(D_{i}\nabla u_{e}^{n}\right)+i_{ion}\left(v^{n},{\text{w}}^{n+1},{\text{c}}^{n+1}\right)=i_{app}^{n+1}. \end{array}$$ (4)

This operator splitting strategy yields at each time step the solution of two large scale linear systems of algebraic equations, arising from the finite element discretization of Eqs (3) and (4), respectively.

In order to ensure parallelization and portability of our Fortran code, we use the PETSc parallel library [37] developed at the Argonne National Laboratory. The two large linear systems at each time step are solved by a parallel conjugate gradient method, preconditioned by the Multilevel Additive Schwarz preconditioner, developed in [38], for the ill-conditioned elliptic system and the Block Jacobi preconditioner for the well conditioned parabolic system. These preconditioners are based on the multilevel PETSc objects PCMG (MultiGrid) with ILU(0) local solvers. The simulations are run on 64 cores of the Linux Cluster INDACO of the University of Milan (sito web) or on 128 cores the Linux cluster GALILEO of the CINECA laboratory (sito web). These computations are quite demanding: each run simulating 2000 ms after delivering a stimulus required about 12 hours on 64 cores, and for each of the 9 settings considered, we carried out several tests with 8 pacing runs and several S1-S4 stimulations as described in the Stimulation protocol section below.

### Computational domain

The domain *H* is the image of a Cartesian periodic slab using ellipsoidal coordinates, yielding a truncated ellipsoid modeling a left ventricle (LV) geometry, described by the parametric equations

$$\left\{ \begin{array}{cc} x=a(r)\;\text{cos}\;\theta \;\text{cos}\;\phi \; & \phi_{min}\leq \phi \leq \phi_{max},\\ y=b(r)\;\text{cos}\;\theta \;\text{sin}\;\phi \; & \theta_{min}\leq \theta \leq \theta_{max},\\ z=c(r)\;\text{sin}\;\theta \; & 0\leq r\leq 1, \end{array}\right.$$

where *a*(*r*) = *a*_1_ + *r*(*a*_2_ − *a*_1_), *b*(*r*) = *b*_1_ + *r*(*b*_2_ − *b*_1_), *c*(*r*) = *c*_1_ + *r*(*c*_2_ − *c*_1_), and *a*_1_ = *b*_1_ = 1.5, *a*_2_ = *b*_2_ = 2.7, *c*_1_ = 4.4, *c*_2_ = 5 (all in *cm*) and *ϕ*_*min*_ = −*π*/2, *ϕ*_*max*_ = 3*π*/2, *θ*_*min*_ = −3*π*/8, *θ*_*max*_ = *π*/8. We will refer to the inner surface of the truncated ellipsoid (*r* = 0) as endocardium and to the outer surface (*r* = 1) as epicardium. In all computations, a structured grid of 512 × 256 × 48 hexahedral isoparametric *Q*_1_ finite elements of size *h* ≈ 0.02 *cm* is used in space, for a total amount of 6447616 mesh nodes The time step size is fixed to *τ* = 0.05 *ms*. Fibers rotate transmurally, linearly with the depth and counterclockwise from epicardium to endocardium, for a total amount of 120^*o*^. The values of the transversely isotropic conductivity coefficients are

$$\begin{array}{cc} \sigma_{l}^{e}=2 & \sigma_{l}^{i}=3\\ \sigma_{t}^{e}=1.3514 & \sigma_{t}^{i}=0.31525, \end{array}$$

all given in *mS*.

### Parameter calibration

#### G406R mutation

According to [8], we consider a heterozygous setting in which the exon 8A containing CaV1.2 protein represents 23% of the total CaV1.2 protein (11.5% wild type (WT) and 11.5% G406R mutant). This setting is denoted by TS-11%. We also consider a second heterozygous setting where, in addition, the exon 8 containing CCaV1.2 protein represents 77% of the total CaV1.2 protein (38.5% WT, 38.5% G406R mutant). This setting is denoted by TS-50%.

Following [39], L-type calcium channels (*I*_*CaL*_) are modeled by

$$I_{CaL}=\left(1-\rho \right)I_{CaL}^{WT}+\rho I_{CaL}^{TS},$$

where $I_{CaL}^{WT}$ are the wild type (WT) channels, $I_{CaL}^{TS}$ are the G406R mutant (TS) channels and *ρ* is the percentage of heterozygosis, thus *ρ* = 0.11 (TS-11%) and *ρ* = 0.5 (TS-50%).

In the TP06 model, the analytic expression of $I_{CaL}^{*}$, with * = *WT*, *TS* is

$$I_{CaL}^{*}=g_{CaL}\;d^{*}\;f^{*}\;f_{2}\;f_{Ca_{ss}}\;4.0\;\left(V-15.0\right)\;\frac{F^{2}}{R\;T}\frac{0.25\;Ca_{ss}\;\text{exp}\;(2.0\;\left(V-15.0\right)\;\frac{F}{R\;T})-Ca_{o}}{\text{exp}\;(2.0\;\left(V-15.0\right)\;\frac{F}{R\;T})-1.0},$$

where *g*_*CaL*_ is the maximal conductance, *Ca*_*ss*_ and *Ca*_*o*_ the subspace and extracellular calcium concentrations, respectively, d* the activation gate, f* *f*_2_ the voltage-dependent inactivation gates, $f_{Ca_{ss}}$ the *Ca*_*ss*_-dependent inactivation gate, *V* the transmembrane potential, *F* the Faraday constant, *R* the gas constant, *T* the absolute temperature. All parameters values are those reported in [31].

The activation function $d_{\infty }^{*}$ is

$$d_{\infty }^{*}=\frac{1.0}{1.0+\text{exp}(\frac{V_{a05}^{*}-V}{S_{a}^{*}})},$$

with activation voltage for 50% channels open $V_{a0.5}^{*}$ and scaling parameter $S_{a}^{*}$ given by

-
$V_{a0.5}^{WT}=-4.0\;mV$ , $S_{a}^{WT}=7.5$;
-
$V_{a0.5}^{TS}=-8.0\;mV$ , $S_{a}^{TS}=7.5$.

The inactivation function $f_{\infty }^{*}$ is

$$f_{\infty }\left(V\right)=\frac{1.0}{1.0+\text{exp}(\frac{V-V_{ina05}^{*}}{S_{ina}^{*}})}$$

with inactivation voltage for 50% channels open $V_{ina0.5}^{*}$ and scaling parameter $S_{ina}^{*}$ given by

-
$V_{ina0.5}^{WT}=-20.0\;mV$ , $S_{ina}^{WT}=7.0$;
-
$V_{ina0.5}^{TS}=0.0\;mV$ , $S_{ina}^{TS}=12.0$.

**Fig 1:**
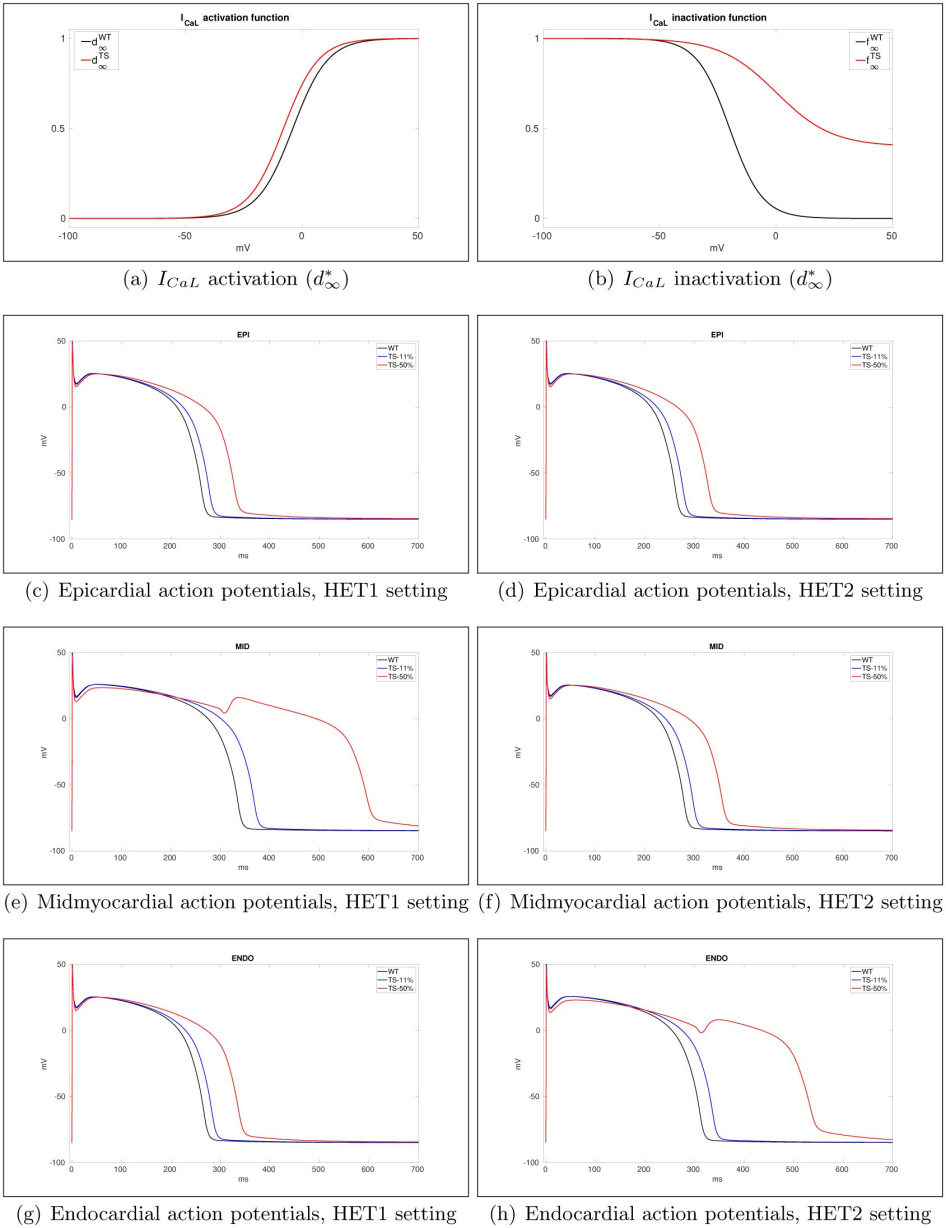
a), b): Wild type (WT) and G406R mutant (TS) *I*_*CaL*_ activation ($d_{\infty }^{*}$) and inactivation ($f_{\infty }^{*}$) functions. Epicardial (c-d), midmyocardial (e-f) and endocardial (g-h) WT, TS-11% and TS-50% action potential waveforms at BCL = 1000 ms in case of HET1 (left) and HET2 (right) transmural heterogeneous settings. The waveforms are computed by solving a one-dimensional version of system (1).

The parameters were adjusted so that our activation ($d_{\infty }^{*}$) and inactivation ($f_{\infty }^{*}$) curves, displayed in Fig 1, match at least qualitatively the experimental plots reported in Fig 4E and 4F of [8], and the resulting effects in our simulated pathological action potential waveforms, also displayed in Fig 1, are comparable with those observed in Fig 6 of [8]. The waveforms in Fig 1 are computed by solving a one-dimensional version of system (1).

#### Transmural heterogeneities of action potential duration

It is well known from in vitro experimental studies [40, 41] that myocytes extracted from different depths of the ventricular wall exhibit different action potential duration (APD). This transmural APD heterogeneity has also been observed in myocardial wedge preparations of different species, see [25, 42–47].

On the basis of that, we model the ventricular tissue with homogeneous (HOM) or transmural heterogeneous distributions of APD. The transmural wall is subdivided into three layers of the same depth: the sub-endocardial layer (ENDO), the midmyocardial layer (MID) and the sub-epicardial layer (EPI). Two types of transmural heterogeneity are considered by scaling the original formulation (given in [31]) of the *I*_*Ks*_ current by different factors:

- in the HET1 setting, the *I*_*Ks*_ current of the ENDO, MID and EPI cells, is multiplied by a factor 1.3, 0.5 and 1.4, respectively. As a result, in this heterogeneous setting, the midmyocardial cells present the longest APD, in agreement with the experimental studies [25, 42, 45–47];
- in the HET2 setting, the *I*_*Ks*_ current of the ENDO, MID and EPI cells is multiplied by a factor 0.7, 1.1 and 1.4, respectively. As a result, in this heterogeneous setting, the sub-endocardial cells present the longest APD, in agreement with the experimental studies [25, 43, 44].

In homogeneous simulation, all cells in the ventricle are assigned the calibration of ENDO cells, with *I*_*Ks*_ multiplied by the factor 1.3. Fig 1 displays the resulting action potential waveforms, computed by solving the one-dimensional cable equation with conductivity coefficient 2 *mS*, whereas Table 1 reports the corresponding APD values at BCL = 1000 ms. Note that in case of TS-50%-HET1 (MID cells) and TS-50%-HET2 (ENDO cells), an EAD occurs.

**Table 1:** Action potential duration of WT and TS endo-, mid- and epicarical cells at BCL = 1000 ms.

	ENDO	MID	EPI
WT-HET1	266	334	261
TS-11%-HET1	282	367	276
TS-50%-HET1	336	596	328
WT-HET2	310	278	261
TS-11%-HET2	336	296	276
TS-50%-HET2	535	355	328

### Simulation setup

We consider the following nine settings:

- WT-HOM: WT membrane properties (*ρ* = 0) without transmural heterogeneities of *I*_*Ks*_;
- TS-11%-HOM: TS membrane properties (*ρ* = 0.11) without transmural heterogeneities of *I*_*Ks*_;
- TS-50%-HOM: TS membrane properties (*ρ* = 0.5) without transmural heterogeneities of *I*_*Ks*_;
- WT-HET1: WT membrane properties (*ρ* = 0) with HET1 transmural heterogeneities of *I*_*Ks*_;
- TS-11%-HET1: TS membrane properties (*ρ* = 0.11) with HET1 transmural heterogeneities of *I*_*Ks*_;
- TS-50%-HET1: TS membrane properties (*ρ* = 0.5) with HET1 transmural heterogeneities of *I*_*Ks*_;
- WT-HET2: WT membrane properties (*ρ* = 0) with HET2 transmural heterogeneities of *I*_*Ks*_;
- TS-11%-HET2: TS membrane properties (*ρ* = 0.11) with HET2 transmural heterogeneities of *I*_*Ks*_;
- TS-50%-HET2: TS membrane properties (*ρ* = 0.5) with HET2 transmural heterogeneities of *I*_*Ks*_.

### Stimulation protocol

Our simulations use the S1—S2—S3—S4 stimulation protocol, a Programmed Ventricular Stimulation (PVS) which in synthesis is the pacing procedure introduced in Brugada et al. in 2003 [48] and frequently used in several experimental studies (see [49] for a recent review and alternative protocols in mice models). In this protocol, a first pacing cycle of several stimulations (S1) at a given cycle length is applied, and then three consecutive extrastimuli (S2, S3, S4) are applied at the closest coupling intervals at which each extrastimulus triggers an arrhythmia. In our case, the first pacing cycle consists of S1 stimuli of 350*mA*/*cm*^3^ for 1 ms on a small area 0.12×0.12×0.06 *cm*^3^ at an endocardial site for the HOM and HET1 setting and an epicardial site for the HET2 setting; the stimulus is delivered at a central location, equidistant from the apex and base of the idealized ventricle, as indicated by the breakthrough (red color) in Fig 7, panel a) at *t* = 380 ms, and in Fig 8, panel a) at *t* = 340 ms. For each simulation setting, we first apply 8 pacing stimuli (S1) at a BCL of 500 ms. Then, a premature stimulus (S2) is delivered 380 ms after S1. If S2 does not generate a reentrant arrhythmia, the S1–S2 coupling interval is shortened in steps of 10 ms until arrhythmia is induced or S2 fails to trigger excitation. If arrhythmia is not induced, an additional S3, and if necessary, an additional S4 stimulus, is delivered in the same manner as S2. The S3 stimulus is initially delivered 350 ms after the previous S2 stimulus, and then shortened until arrhythmia is induced or the stimulus fails to induce arrhythmia. Analogously for S4. The criterion adopted for successful arrhythmia induction is the onset of a reentrant excitation that remains sustained up to the end of simulation time, set to 4000 ms after the last stimulus.

This stimulation protocol is quite demanding computationally, since each run simulating 2000 ms after delivering a stimulus requires about 12 hours on 64 cores of our Linux clusters. This cost, scaled by the simulated time, occurs for each of the 8 pacing runs and S1-S2-S3-S4 stimulations, which we repeated in each of the 9 settings considered, from WT-HOM to TS-50%-HET2 (see previous section).

### Post-processing

In each simulation, we save the time evolution of the transmembrane potential on a matrix of 64 × 33 exploring multi-electrode transmural needle, equally distributed through the LV. Each needle carries 7 recording sites, equally spaced along the transmural epi-to-endocardial direction. Therefore, we have a total of 14784 recording sites.

From each transmembrane potential waveform, we compute:

- the activation time (ACTI), defined as the instant of maximal slope during the depolarization phase of the action potential;
- the repolarization time (REPO), defined as the instant of minimal slope during the repolarization phase of the action potential;
- the action potential duration (APD), defined as the difference APD = REPO-ACTI.

As a byproduct, we compute the following quantities:

- total activation time dispersion (*dAT*_*tot*_), defined as *dAT*_*tot*_ = max(ACTI)-min(ACTI), where max and min are taken over all the 14784 recording sites;
- total repolarization time dispersion (*dRT*_*tot*_), defined as *dRT*_*tot*_ = max(REPO)-min(REPO), where max and min are taken over all the 14784 recording sites;
- average transmural repolarization dispersion (*dRT*_*trans*_), defined as the average over all the 64 × 33 transmural needles of max(REPO)-min(REPO), where max and min are taken over the 7 sites of each needle;
- total APD dispersion (*dAPD*_*tot*_), defined as *dAPD*_*tot*_ = max(APD)-min(APD), where max and min are taken over all the 14784 recording sites;
- average transmural APD dispersion (*dAPD*_*trans*_), defined as the average over all the 64 × 33 transmural needles of max(APD)-min(APD), where max and min are taken over the 7 sites of each needle

## Numerical results

We have simulated the electrical activity of the idealized LV model, considering the six settings described above in the Simulation setup section. For each setting, we first perform 8 beats at a BCL = 500 ms. Then we apply an S1-S2-S3-S4 stimulation protocol by reducing the coupling interval between subsequent stimulations. In HOM and HET1 simulations, the stimulus is applied at an endocardial site, whereas in HET2 simulations the stimulus is applied at an epicardial site.

### Total and transmural dispersion of ACTI, REPO, APD

In Table 2, we report the *dAT*_*tot*_, *dRT*_*tot*_, *dRT*_*trans*_, *dAPD*_*tot*_ and *dAPD*_*trans*_ parameters, as computed after the S1 stimulus, thus corresponding to a BCL = 500 ms. In the settings without transmural heterogeneities (WT-HOM, TS-11%-HOM, TS-50%-HOM), we do not observe significant differences between the pathological settings (TS-11% and TS-50%) and the WT setting, in terms of all five parameters. On the other hand, in the settings with transmural heterogeneities, we do observe a strong increase of total and transmural dispersion of repolarization and APD in case of TS-50%-HET1 with respect to WT-HET1 (331 vs 195 ms for *dRT*_*tot*_, 94 vs 25 ms for *dRT*_*trans*_, 144 vs 39 ms for *dAPD*_*tot*_, 97 vs 27 ms for *dAPD*_*trans*_) and in case of TS-50%-HET2 with respect to WT-HET2 (382 vs 218 ms for *dRT*_*tot*_, 145 vs 32 ms for *dRT*_*trans*_, 196 vs 42 ms for *dAPD*_*tot*_, 142 vs 31 ms for *dAPD*_*trans*_).

**Table 2:** Total activation time dispersion (*dAT*_*tot*_), total repolarization time dispersion (*dRT*_*tot*_), average transmural repolarization dispersion (*dRT*_*trans*_), total APD dispersion (*dAPD*_*tot*_), average transmural APD dispersion (*dAPD*_*trans*_), as defined in the post-processing section, computed after the S1 stimulus. All parameters are reported in ms.

Simulation	*dAT*_*tot*_	*dRT*_*tot*_	*dRT*_*trans*_	*dAPD*_*tot*_	*dAPD*_*trans*_
WT-HOM	209	196	9 ± 6	16	4 ± 1
TS-11%-HOM	211	197	9 ± 6	16	4 ± 1
TS-50%-HOM	221	208	10 ± 7	17	4 ± 1
WT-HET1	194	195	25 ± 6	39	27 ± 6
TS-11%-HET1	196	202	30 ± 8	46	33 ± 7
TS-50%-HET1	220	331	94 ± 20	144	97 ± 19
WT-HET2	200	218	32 ± 11	42	31 ± 3
TS-11%-HET2	202	228	40 ± 11	52	40 ± 4
TS-50%-HET2	223	382	145 ± 52	196	142 ± 46

The results have also shown that, in the settings without transmural heterogeneities, the average APD on the entire LV increases from 267 ms (WT-HOM) to 286 ms (TS-11%-HOM) and 346 ms (TS-50%-HOM), whereas, in the settings with transmural heterogeneities, it increases from 277 ms (WT-HET) to 297 ms (TS-11%-HET) and 408 ms (TS-50%-HET). This latter increase is mainly due to the increase of APD in the midmyocardial layers.

**Fig 2:**
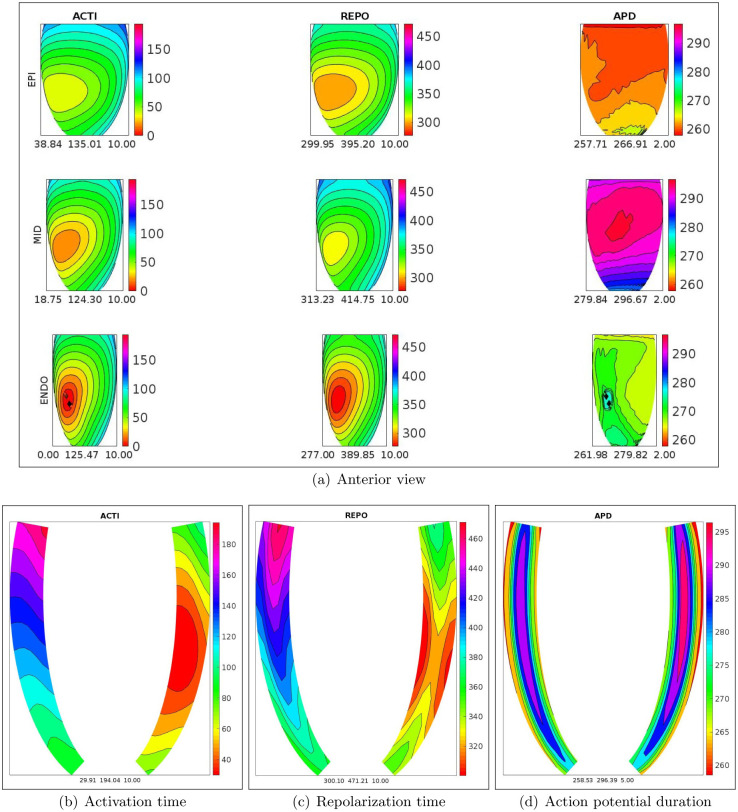
WT-HET1 simulation, S1 stimulus. a): spatial distributions of activation time (ACTI), repolarization (REPO) and action potential duration (APD) on the endocardial, midmyocardial and epicardial sections. Transmural longitudinal sections of spatial distributions of b) activation time (ACTI), c) repolarization (REPO) and d) action potential duration (APD).

**Fig 3:**
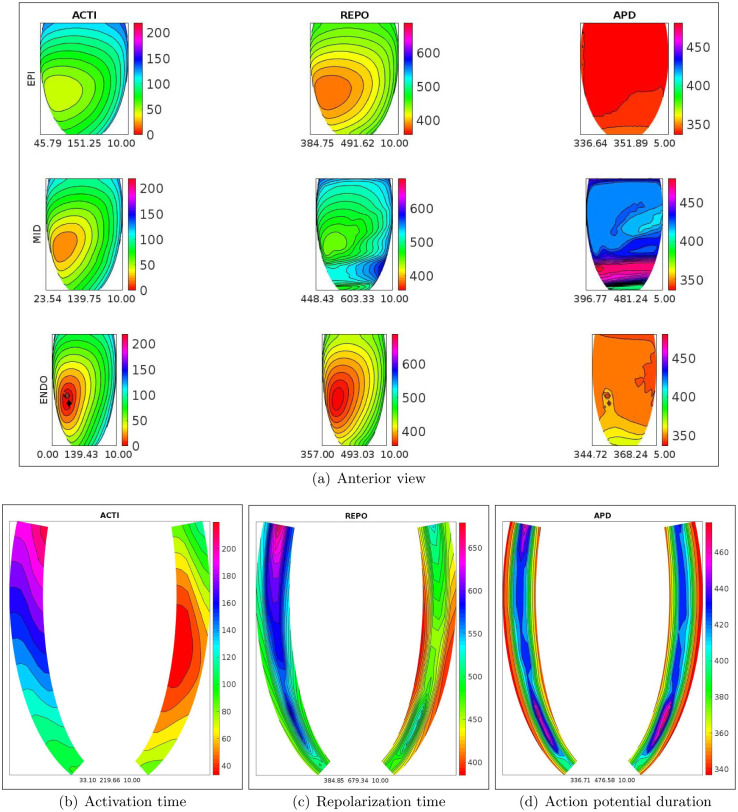
TS-50%-HET1 simulation, S1 stimulus. a) spatial distributions of activation time (ACTI), repolarization (REPO) and action potential duration (APD) on the endocardial, midmyocardial and epicardial sections. Transmural longitudinal sections of spatial distributions of b) activation time (ACTI), c) repolarization (REPO) and d) action potential duration (APD).

In order to better understand the dynamics of the activation and repolarization processes, Figs 2 and 3, display the spatial distributions of ACTI, REPO, APD, computed after the S1 stimulus, on the epicardial, midmyocardial, endocardial and on two longitudinal transmural sections, for the WT-HET1 and TS-50%-HET1 settings. Note that, on the midmyocardial section, TS-50%-HET1 presents a strong increase of REPO and APD dispersion with respect to WT-HET1 (240 vs 158 ms for REPO, 85 vs 17 ms for APD). These dispersions are computed as the difference between the maximum and minimum value of the corresponding distribution (REPO or APD), on the midmyocardial section.

**Fig 4:**
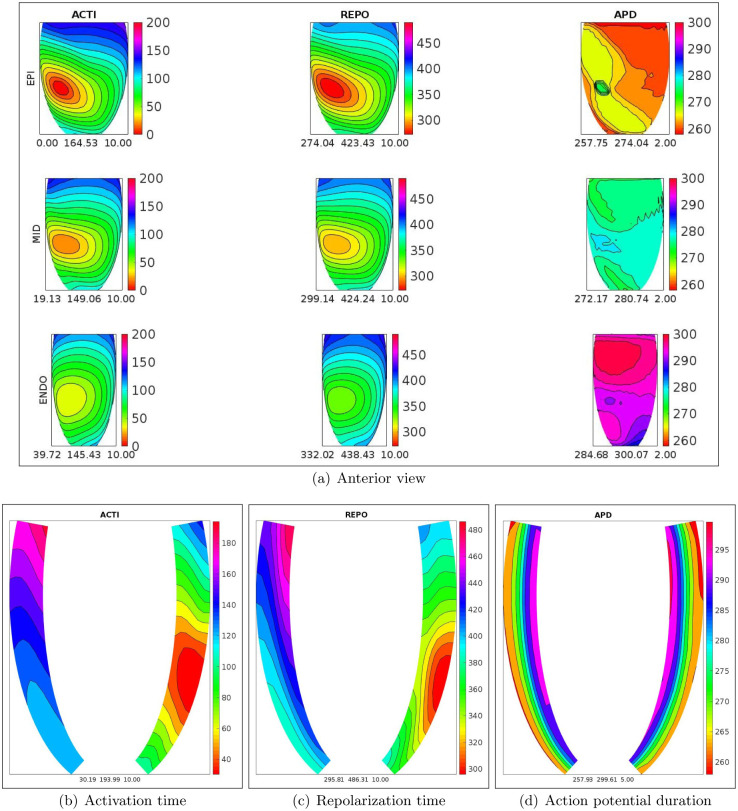
WT-HET2 simulation, S1 stimulus. a) Spatial distributions of activation time (ACTI), repolarization (REPO) and action potential duration (APD) on the endocardial, midmyocardial and epicardial sections. Transmural longitudinal sections of spatial distributions of b) activation time (ACTI), c) repolarization (REPO) and d) action potential duration (APD).

**Fig 5:**
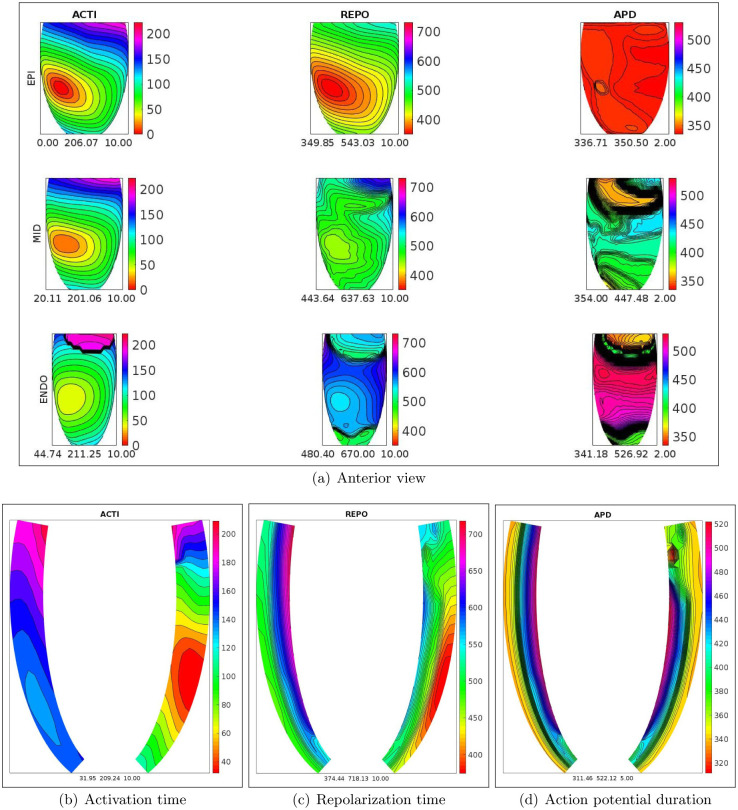
TS-50%-HET2 simulation, S1 stimulus. a) Spatial distributions of activation time (ACTI), repolarization (REPO) and action potential duration (APD) on the endocardial, midmyocardial and epicardial sections. Transmural longitudinal sections of spatial distributions of b) activation time (ACTI), c) repolarization (REPO) and d) action potential duration (APD).

Analogously, Figs 4 and 5, report the spatial distributions of ACTI, REPO, APD for the WT-HET2 and TS-50%-HET2 settings. In this case, on the endocardial section, TS-50%-HET2 presents a strong increase of REPO and APD dispersion with respect to WT-HET2 (252 vs 158 ms for REPO, 189 vs 16 ms for APD).

### Arrhythmogenesis with S1-S2-S3-S4 stimulation protocol

**Fig 6:**
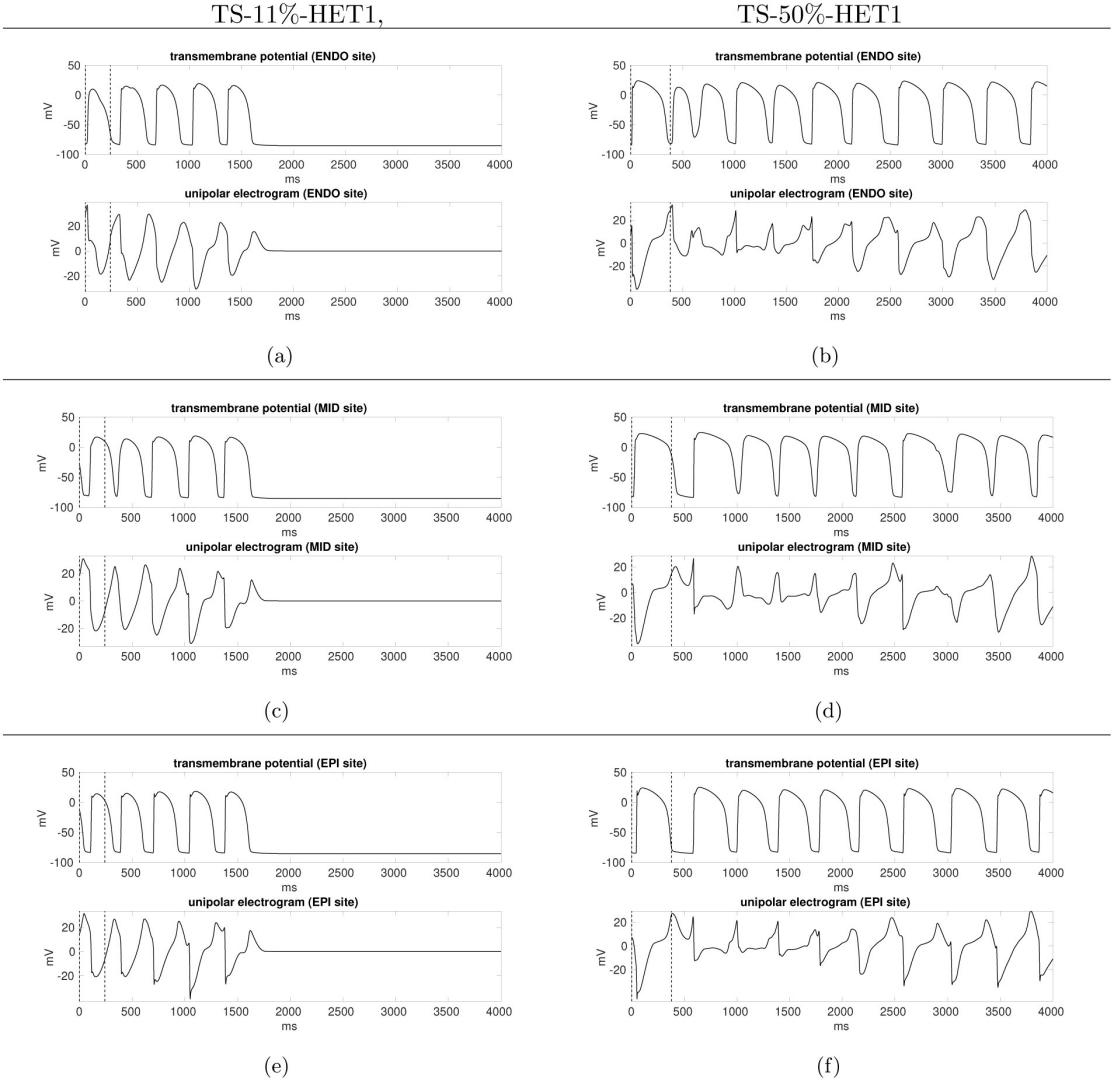
TS-11%-HET1 simulation (left), TS-50%-HET1 simulation (right). Action potential waveforms and unipolar electrogram at endocardial (a-b), midmyocardial (c-d) and epicardial (e-f) sites, located at the central region of the LV. *t* = 0 ms is the S3 stimulus. The vertical dashed line indicates the S4 stimulus, applied after an S3-S4 coupling interval of 240 ms.

In the TS-11%-HET1 setting, after the S1 stimulus, we apply an S2 stimulus with an S1-S2 coupling interval of 320 ms, and an S3 stimulus with an S2-S3 coupling interval of 260 ms. After the S4 stimulation delivered at an S3-S4 coupling interval of 240 ms, a conduction block occurs when the excitation wavefront reaches the midmyocardial region, because this latter region presents a larger APD and thus effective refractory period than the endo- and epicardial regions. Then the electrical signal proceeds around the region of block, towards epicardium. After having excited the epicardial surface, the excitation wavefront comes back into the midmyocardial regions, now excitable again, generating a reentrant ventricular tachycardia, which dies after three cycles of reentry, as confirmed by the transmembrane potential and unipolar electrogram waveforms reported in Fig 6 (see also S1 Movie in the supplementary data).

**Fig 7:**
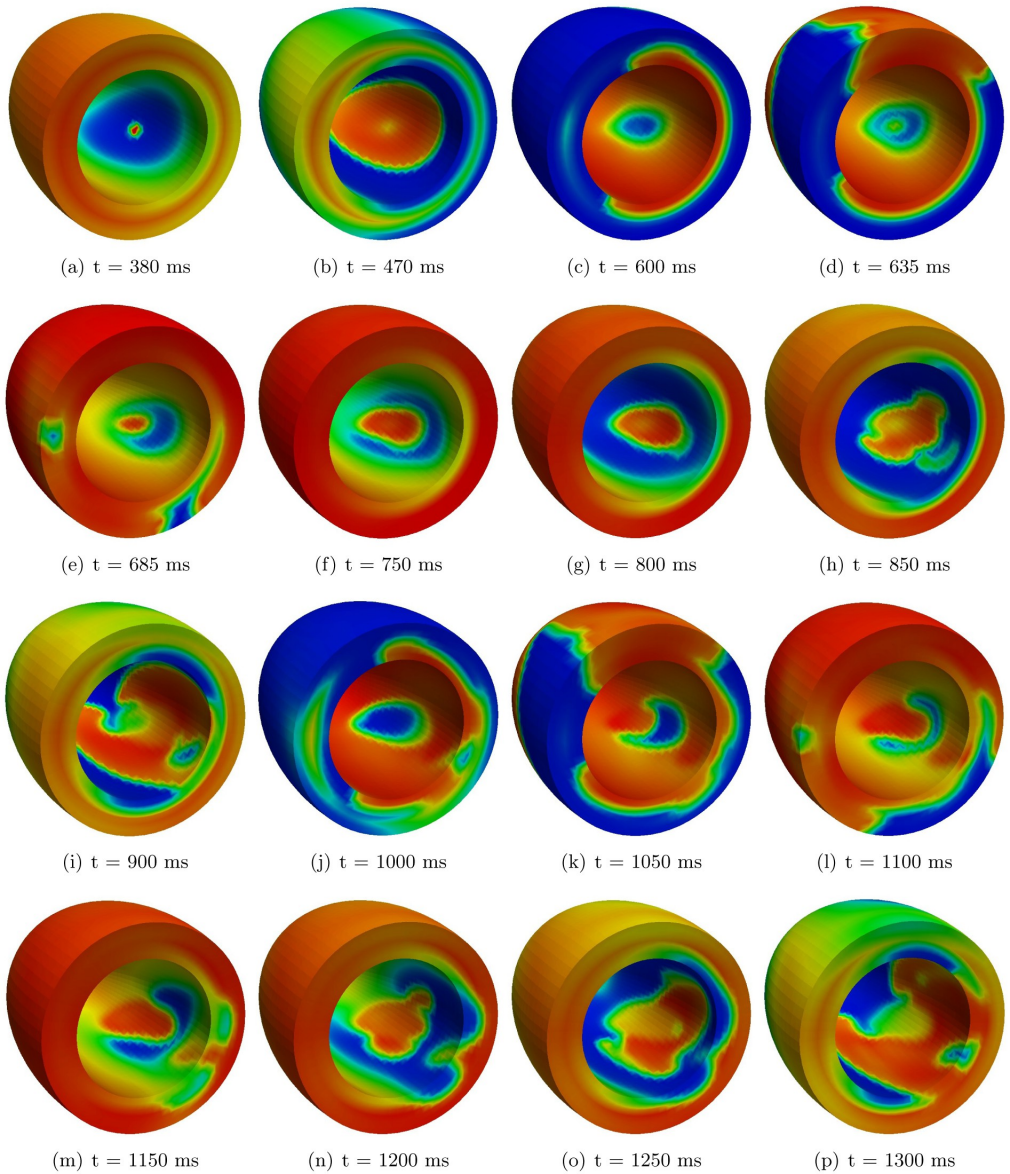
TS-50%-HET1 simulation. Transmembrane potential snapshots. *t* = 0 corresponds to the S1 stimulus.

In the TS-50%-HET1 setting instead, after the S2 stimulation delivered at an S1-S2 coupling interval of 380 ms (Fig 7(a)), we do not observe an epicardial breakthrough (BKT), since the spread of excitation through the transmural wall is blocked, due to the refractoriness induced by the APD heterogeneity of the midmyocardial cells. Therefore, the epicardial surface is activated later by a wavefront propagating from the apex towards the base. Then, excitation propagates across the wall from the sub-epicardial and midmyocardial regions towards the endocardium, yielding a BKT inside a region around the stimulation area (Fig 7(d)). The endocardial BKT triggers the first cycle of reentry, which starts propagating slowly since the region has only partially recovered (Fig 7(e) and 7(f)). The reentrant excitation wavefront, constituted by two scroll waves, is maintained for about five cycles, generating a reentrant ventricular tachycardia (Fig 7(h)–7(p)). Then, although one of the two scroll wave dies, the reentry remains sustained, as confirmed by the transmembrane potential and unipolar electrogram waveforms reported in Fig 6 (see also S2 Movie in the supplementary data).

**Fig 8:**
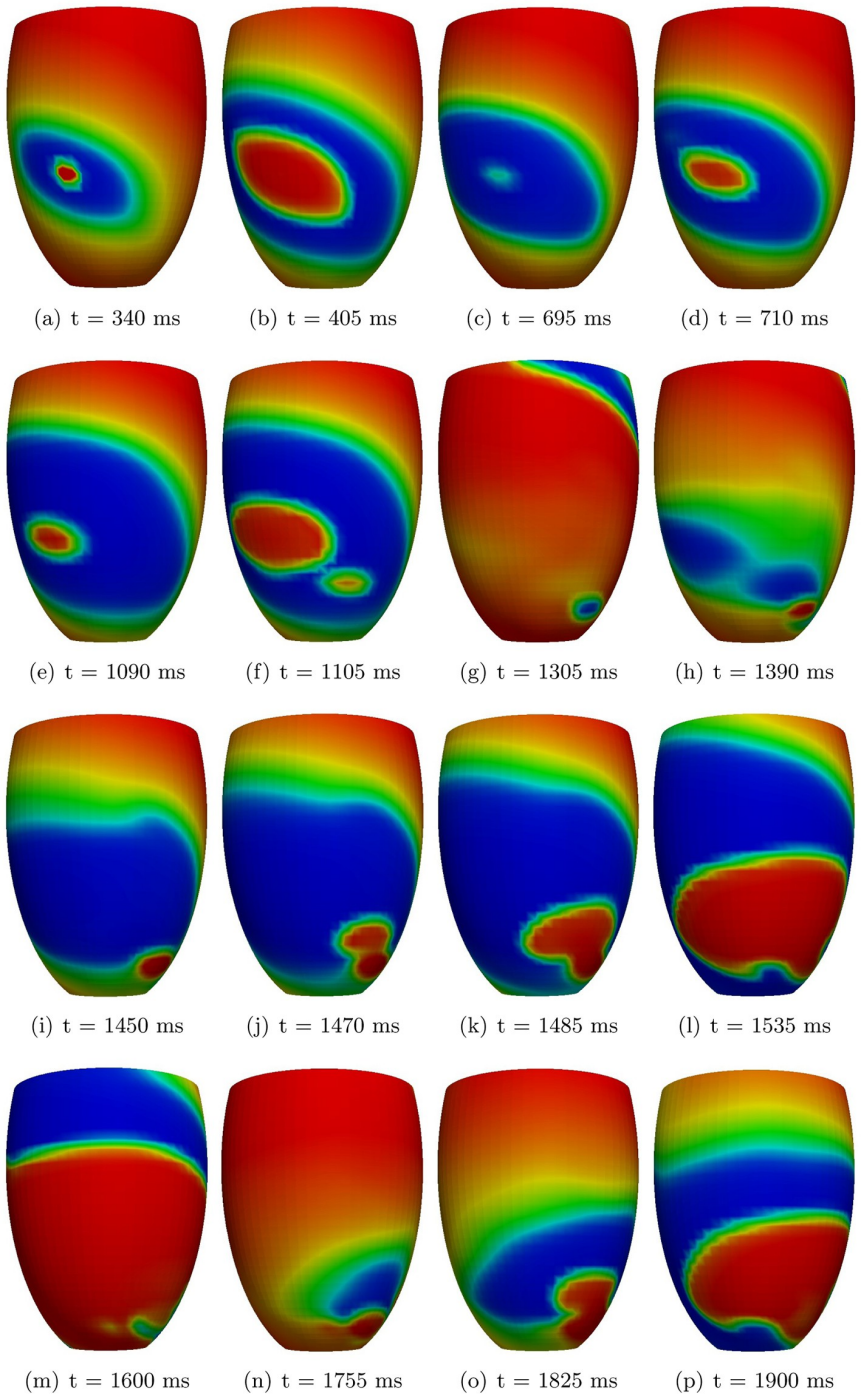
TS-50%-HET2 simulation. Transmembrane potential snapshots. *t* = 0 corresponds to the S2 stimulus.

In the TS-50%-HET2 setting, after the S1 stimulus, we apply an S2 stimulus with an S1-S2 coupling interval of 360 ms. After the S3 stimulation delivered at an S2-S3 coupling interval of 340 ms (Fig 8(a)), a conduction block occurs when the excitation wavefront reaches the subendocardial region, which presents a larger APD and effective refractory period than the midmyocardial and sub-epicardial regions. Then the electrical signal proceeds around the region of block, towards the endocardium. After having excited the endocardial surface, the excitation wavefront comes back into the midmyocardial regions, now excitable again, yielding an epicardial BKT (Fig 8(c)), at about 360 ms after the S3 stimulus, and thus triggering the first cycle of reentrant excitation. After having excited the epicardium, the reentrant wavefront moves towards the endocardium, reactivating first the apical regions. The activation wavefront moves again back to epicardium, yielding two BKTs (Fig 8(e) and 8(f)). Then, at about 1050 ms after the S3 stimulus, a scroll wave originates (Fig 8(h)–8(p)), in the apical sub-epicardial region, determining the maintenance of reentry and thus generating a sustained ventricular tachycardia (see S3 Movie in the supplementary data).

In the WT-HET1, WT-HET2, TS-11%-HET2 settings and in all the settings without transmural heterogeneities, no conduction blocks occur during the entire S1-S2-S3-S4 stimulation protocol. Consequently reentry is not induced in these cases.

## Discussion

We have investigated by computer modeling the role played by transmural APD heterogeneity on the onset of reentrant ventricular arrhythmias in the presence of LQT8 syndrome. This congenital heart disease might be produced by several mutations in the CACNA1C gene, encoding the CaV1.2 subunit of the ICaL current. The mutation considered here is the G406R mutation [7, 8].

The three-dimensional numerical simulations reported are based on the Bidomain model of electrocardiology, coupled with the TP06 membrane model, taking into account the fiber anisotropy of the ventricular tissue. Our calibration of the G406R mutation in the ICaL current provided by the TP06 model is in agreement with the experimental and computational findings reported in [8].

We have considered two types of transmural APD heterogeneity: in the first one (HET1), midmyocardial cells (M cells) present a prolonged APD with respect to sub-endocardial and sub-epicardial cells; in the second one (HET2), sub-endocardial cells present a prolonged APD with respect to midmyocardial and sub-epicardial cells.

The results have shown that, with both HET1 and HET2 APD heterogeneities, the presence of G406R mutation with 50% of heterozygosis (TS-50%) yields a significant increase of transmural dispersion of repolarization. This growth is due to the prolonged APD of the midmyocardial and sub-endocardial cells in the HET1 and HET2 settings, respectively.

During the implementation of the S1-S2-S3-S4 stimulation protocol, the high transmural repolarization gradients occurring in the TS-50%-HET1 and TS-50%-HET2 simulations cause conduction blocks of the excitation wavefronts elicited by premature beats. Such blocks of conduction induce the onset of reentrant ventricular tachycardia. EADs occuring in the regions of prolonged APD facilitate the maintenance of the cycles of reentry, as observed in previous computational studies [50, 51] for other LQT variants. When transmural APD heterogeneities are neglected, in WT-HOM, TS-11%-HOM and TS-50%-HOM simulations, reentry does not occur, irrespective of the presence of LQT8 pathology. The onset of reentry due to dispersion of repolarization after premature beats and the occurrence of EADs was also observed in the recent experimental study [10]. However, in [10], the mechanism responsible for the increase of dispersion of repolarization was an activation delay due to impairment of *I*_*Na*_ current, rather than the combination of G406R mutation and intrinsic APD heterogeneities.

We recall that in our HET1 heterogeneous setting, midmyocardial cells (M cells) present a prolonged APD. In vitro experimental studies have demonstrated that isolated myocytes, extracted from different depths across the ventricular wall, exhibit transmembrane action potentials with different durations, see [40, 41], with the APD of M cell being longer than that of sub-endocadial and sub-epicardial cells. This transmural APD heterogeneity has also been observed in myocardial wedge preparations, see [25, 42, 45–47]. Despite these findings obtained in myocardial in vitro and wedge preparations, high transmural repolarization gradients have never been observed in intact hearts, see e.g. [52–55]. It has been suggested that the electrotonic coupling present in vivo mask cellular heterogeneities, yielding a reduced dispersion of repolarization times and APDs. Indeed in our WT-HET1 simulation, the resulting transmural dispersion of repolarization time and APD is not large (25–32 ms for repolarization time and 39–42 ms for APD), and it is comparable with those observed in intact hearts.

In our HET2 heterogeneous setting instead, an epi-to-endo transmural APD gradient is introduced, with sub-endocardial cells presenting a prolonged APD. This setting is in agreement with recent experimental studies on human wedge preparations reported in [25, 43, 44].

### Limitations

In our model of *I*_*CaL*_ current, we have assumed that the G406R mutation affects only the voltage dependent inactivation gate. However, experiments reported in the recent study [39] have shown that the G406R mutation might affect also the calcium dependent inactivation gate of the *I*_*CaL*_ current. Future tissue simulation studies should investigate how the G406R mutation in the *I*_*CaL*_ calcium dependent inactivation gate influences the onset of arrhythmic events. Moreover, a future computational study should also take into account the reduction of *I*_*Na*_ current, as suggested in the experimental work [10].

## Supporting information

S1 Movie Movie (file name TS_0115_S4_240.avi) of TS-11%-HET1 simulation. S3 stimulus is given at t = 0 ms. S4 stimulus is given 240 ms after S3. S4 stimulus triggers reentry, which is not sustained.
(AVI)

S2 Movie Movie (file name TS_05_S2_380.avi) of TS-50%-HET1 simulation. S1 stimulus is given at t = 0 ms. S2 stimulus is given 380 ms after S1. S2 stimulus triggers reentry, which is sustained.
(AVI)

S3 Movie Movie (file name TS_05_het2_S3_340.avi) of TS-50%-HET2 simulation. S2 stimulus is given at t = 0 ms. S3 stimulus is given 340 ms after S2. S3 stimulus triggers reentry, which is sustained.
(AVI)
